# Characteristics of Merging Plasma Plumes for Materials Process Using Two Atmospheric Pressure Plasma Jets

**DOI:** 10.3390/ma17194928

**Published:** 2024-10-09

**Authors:** Sang Un Jeon, Jae Wan Kim, Hyun-Young Lee, Gyoo-Cheon Kim, Hae June Lee

**Affiliations:** 1 Department of Electrical Engineering, Pusan National University, Busan 46241, Republic of Korea; un97708@pusan.ac.kr (S.U.J.); 1223kjw@pusan.ac.kr (J.W.K.); 2 Research and Development Team, Feagle Co., Ltd., Yangsan 50561, Republic of Korea; leehyunyoung@naver.com; 3 Department of Anatomy and Cell Biology, School of Dentistry, Pusan National University, Yangsan 50612, Republic of Korea; ki91000m@pusan.ac.kr

**Keywords:** atmospheric pressure plasma jet, plasma plume merging, Schlieren imaging, optical emission spectroscopy

## Abstract

Atmospheric pressure plasma jets (APPJs) have attracted significant attention due to their ability to generate plasma without vacuum systems, facilitating their use in small areas of plasma processing applications across various fields, including medicine, surface treatment, and agriculture. In this study, we investigate the interaction between two helium plasma jets, focusing on the effects of varying flow rate, voltage, and directional angle. By examining both in-phase and out-of-phase configurations, this research aims to elucidate the fundamental mechanisms of plasma plume merging, which has critical implications for optimizing plasma-based material processing systems. We demonstrate that while increasing voltage and flow rate for the in-phase condition leads to an extended plasma plume length, the plumes do not merge, maintaining a minimal gap. Conversely, plasma plume merging is observed for the out-of-phase condition, facilitated by forming a channel between the jets. This study further explores the impact of these merging phenomena on plasma chemistry through optical emission spectroscopy, revealing substantial differences in the emission intensities of OH, the second positive system of $N_{2}$, and the first negative system of $N_{2}^{+}$. These findings offer valuable insights into controlling plasma jet interactions for enhanced efficiency in plasma-assisted processes, particularly where plume merging can be leveraged to improve the treatment area and intensity.

## 1. Introduction

Atmospheric pressure plasma can generate plasma without vacuum equipment, such as chambers or pumps. Owing to this advantage, it has been applied in the medical field for cancer treatment and wound disinfection [1,2,3]. In surface treatment, it has been used for coating and etching [4,5,6], while in agriculture, it has been utilized for seed-promoting growth and removing contaminants [7,8,9,10]. Furthermore, in material processing, it has been employed for surface modification, deposition, and micro-milling [11,12,13]. These examples demonstrate that atmospheric pressure plasma has been widely studied and applied across various fields for several decades. Among the various applications, atmospheric pressure plasma jets (APPJs) are particularly noteworthy. APPJs typically utilize noble gases (e.g., He, Ar) as working gases, either in their pure form [14,15,16,17,18] or as mixtures with small amounts of other gases, such as N_2_ or O_2_ [19,20,21,22,23]. Plasma characteristics vary depending on the type of working gas and the type and mole fraction of the added gases. Additionally, while APPJs have a relatively simple structure, variations in plasma properties can arise due to differences in structure [24,25] and the applied voltage waveform [26,27]. Consequently, many laboratories customize plasma jets to suit the specific requirements of their application fields.

Plasma jets are typically used either as a single jet for plasma treatment in a small area or as a plasma jet array, where multiple jets are arranged to expand the treatment area. In plasma jet arrays, jet-to-jet interactions occurred. Kim et al. [28] observed that in a honeycomb-structured plasma jet array, with increasing flow rate under constant voltage, the optical intensity of the central plasma jet transitions from an intense plasma mode characterized by strong optical emission from the central jet to a well-collimated plasma mode. Furthermore, Wan et al. [29] investigated the variation in plume deflection angle in a plasma jet due to jet-to-jet interactions by arranging three plasma jets horizontally. Their findings indicated that the deflection angle decreases with increasing flow rate and increases with higher applied voltage. These characteristics can be leveraged to enhance the plasma processing area. In the case of counter-propagating plasma jets, the interaction between the jets resulted in distinct characteristics. Douat et al. [30,31] observed that when the applied voltage is in phase, the propagation velocity decreases due to the interaction between plasma bullets from the two jets, preventing the bullets from passing through each other. Additionally, they identified a unique phenomenon termed “pink glow”, attributed to a secondary glow discharge. Cho et al. [32] measured position-specific variations in electrical potential using a high-voltage probe when two plasma jets were subjected to either in-phase or out-of-phase voltage. According to M.J. Johnson et al. [33,34], the power consumption, electron density, and emission spectra were measured when a phase difference existed between two plasma jets. The study revealed that the number of high-energy electrons increased with larger phase shifts, and there were differences in plasma characteristics depending on the presence of a substrate. The total power consumption decreased as the phase shift increased. Furthermore, when a 3D material was placed between two counter-propagating plasma jets, it was confirmed that plasma treatment on the material surface was possible through phase difference control. These findings indicate the potential application of two plasma jets in surface treatment processes.

As each jet can be controlled with a different gas mixture and driving voltage, a two-plasma jet system is adequate for materials surface treatment, especially when various chemical reactions are required on the surface. Moreover, the surface interactions are not only limited to chemical reactions but also includes physical reactions because the speed of the plasma bullet from the plasma jet reaches from 10^4^ up to 10^5^ m/s [35]. In this paper, we investigated the changes in the physical properties of the plasma plumes generated by two plasma jet interactions using Schlieren imaging and digital photography, varying flow rate, voltage, and directional angle between the jets at 90°, 135°, and 180°. Additionally, optical emission spectroscopy (OES) was employed to examine the changes in spectroscopic properties resulting from the interaction between the two plasma jets.

## 2. Materials and Methods

**Figure 1:**
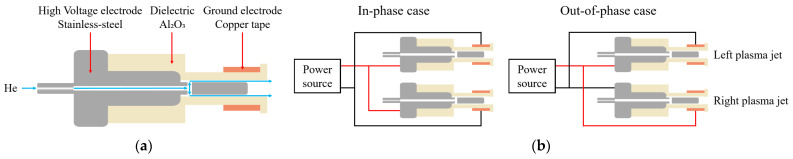
Schematics of (**a**) the plasma jet device and (**b**) in-phase and out-of-phase cases.

Figure 1a shows a schematic diagram of a coaxial dielectric barrier discharge (DBD) plasma jet. The electrode of the plasma jet is stainless steel, with helium (He) gas injected through a 1 mm internal diameter orifice. The stainless steel electrode is encased in an alumina ceramic tube coaxially aligned with the electrode. The outer and inner diameters of the alumina ceramic tube are 6 mm and 4 mm, respectively, while the stainless steel electrode rod has an inner diameter of 3 mm. The discharge gap between the stainless steel electrode and the alumina tube is 0.5 mm. To secure the electrode positioning, a 10 mm wide copper tape is affixed to the alumina tube. The total length of the plasma jet is 40 mm. For plasma generation, helium gas with a purity of 99.999% is injected through a mass flow meter (VIC-D220, Korea Instruments T&S, Seoul, Republic of Korea) at a flow rate ranging from 2 to 4 L per minute (LPM).

A homemade amplifier was connected to a DC power supply (P3030D, ADV@NTEK, Busan, Republic of Korea) to generate a sinusoidal high-voltage (HV) waveform. The amplifier’s output voltage amplitude ranges from 0 to 2.5 kV. Voltage and current waveforms were measured using a high voltage probe (PPE20KV, LeCroy, New York, NY, USA) and a current monitor (2877, Pearson Electronics Inc., Palo Alto, CA, USA), respectively, both connected to an oscilloscope (TDS 2024C, Tektronix, Beaverton, OR, USA). This experiment utilized a sinusoidal voltage source with an amplitude of 1.5 to 2.5 kV and a frequency of 16 kHz. Figure 1b illustrates the circuit configurations for the in-phase and out-of-phase cases. When the HV is applied to the stainless steel and the ground potential is applied to the copper tape relative to the left plasma jet, it is designated as the in-phase case. Conversely, when the potentials are applied oppositely, it is called the out-of-phase case. We fixed the left plasma jet in the horizontal direction and varied the position of the right plasma jet, placing it at 90°, 135°, and 180°.

**Figure 2:**
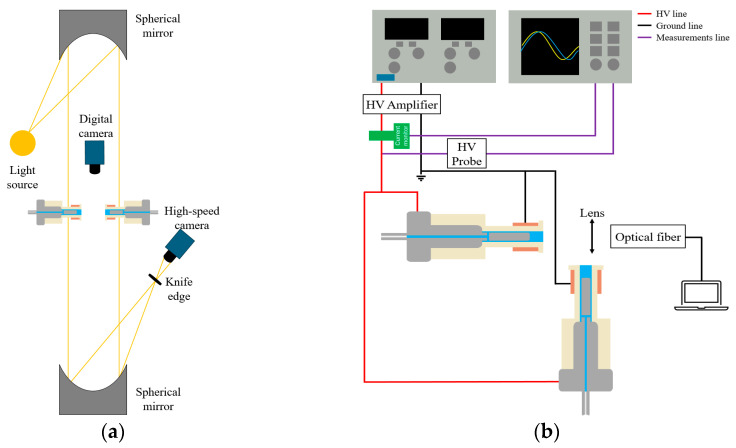
Schematic diagrams of the (**a**) Schlieren and (**b**) OES systems.

Figure 2a presents a schematic diagram of the Schlieren imaging setup. A standard Z-type Schlieren imaging system was employed [36]. The distance between the two spherical mirrors was fixed at 1.52 m, with the plasma jet positioned at the midpoint between the mirrors. A knife edge was placed in front of a high-speed camera (C211, Phantom, NJ, USA) to capture the Schlieren images. Figure 2b presents a schematic diagram of the optical emission spectroscopy (OES) system setup. Due to measurement constraints, the two plasma jets were arranged at a 90° angle to facilitate the measurement of emission intensity from the tip of the left plasma jet. An OES device (SR4, Ocean Insight, Orlando, FL, USA) was used to measure the emission within the wavelength range of 300–750 nm. The exposure time was set to 5 s, and the data were averaged over 15 measurements. The distance between the plasma jet tip and the optical fiber was 50 mm.

## 3. Results

### 3.1. Electrical Diagnosis

**Figure 3:**
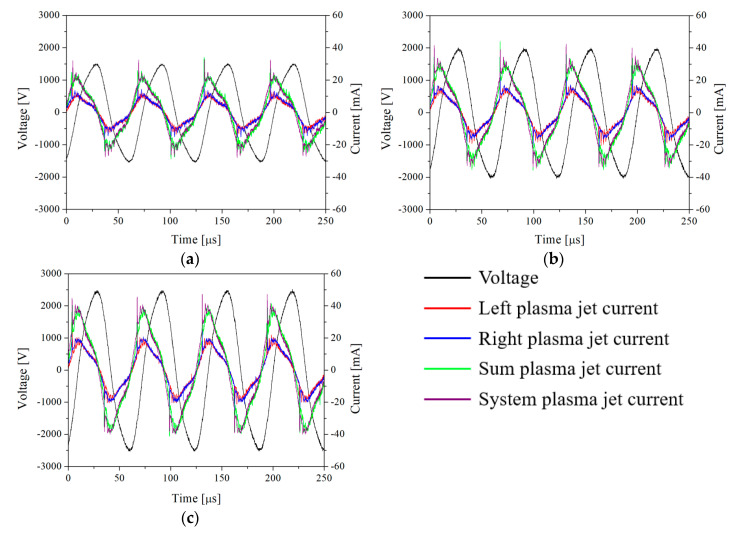
The voltage–current waveforms of He plasma jets with applied voltages of (**a**) 1.5 kV, (**b**) 2 kV, and (**c**) 2.5 kV.

Figure 3 presents the voltage–current waveforms when the voltages of 1.5, 2, and 2.5 kV are applied to the two plasma jets. The voltage was measured across the entire system using a high-voltage probe connected to an oscilloscope, as shown in Figure 2b. Frequency was fixed at 16 kHz for all cases. The current waveforms were measured using a current monitor connected to an oscilloscope, as illustrated in Figure 2b. As shown in Figure 1b, the red line represents the current measured from the left plasma jet, while the blue line represents the current measured from the right plasma jet. The green line shows the combined current from both the left and right plasma jets, and the purple line represents the current measured across the entire system.

### 3.2. Discharge Images

**Figure 4:**
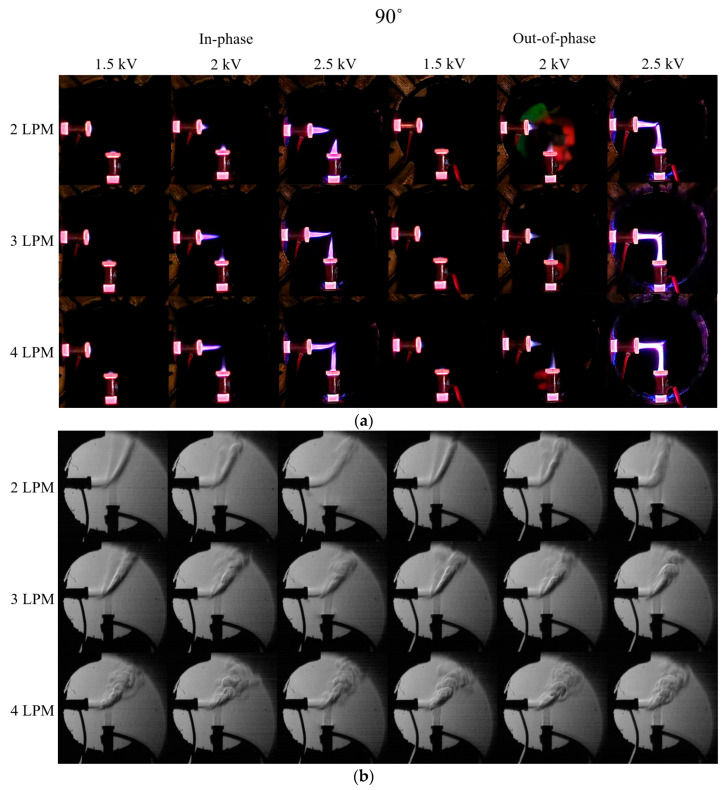
(**a**) Digital camera images and (**b**) Schlieren images for in-phase and out-of-phase configurations with two plasma jets positioned at 90°.

Figure 4 presents the digital camera and Schlieren images for the 90° in-phase and out-of-phase cases. When 1.5 kV is applied in the 90° case, the digital camera images showed minimal plasma plume formation in both the in-phase and out-of-phase cases. Additionally, it was observed that the plasma plume length increases with rising voltage and flow rate. When a voltage of 2.5 kV was applied at 4 LPM for the in-phase case, the ends of the plasma plumes did not merge and appeared to float upward. Analysis of the Schlieren and digital camera images suggested that this was due to the influence of the He flow from both plasma jets and the repulsion between the two plasma plumes. In contrast, when a voltage of 2.5 kV was applied for the out-of-phase case, the plasma plumes from the two jets merged.

**Figure 5:**
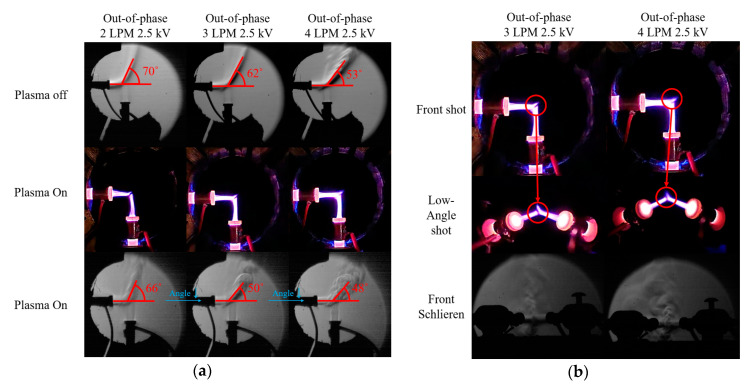
(**a**) Digital camera images and Schlieren images of the out-of-phase case with 0 kV (top) and 2.5 kV (middle, bottom) applied at 2–4 LPM, and (**b**) low-angle shots and Schlieren images of the two plasma jets at a 90° out-of-phase case with 2.5 kV applied at 3 and 4 LPM.

Figure 5a shows the effect of the gas flow rate on the interaction angle of the two jets for the out-of-phase case at 0 kV (plasma off, top) and 2.5 kV (plasma on, middle, and bottom). Unlike the case at 2 LPM, where no merging occurred, two plasma plumes merge at 3 or 4 LPM, as shown in Figure 4. Schlieren images revealed that the collision angle between the flows generated by the two plasma jets decreased from approximately 70° to 53° in the plasma-off case and from approximately 66° to 48° in the plasma-on case. The measurement uncertainty was ±6°. In the plasma-off case, the angle decreased linearly, while in the plasma-on case, there was a significant difference in the angle reduction before and after plume merging. This indicates that plume merging occurs as the collision angle between the two flows decreases, and angle reduction is influenced by both flow rate and plasma presence. Although not detailed in this paper, it was also observed that plume merging can occur in the 2 LPM case when external fluctuations are present, causing the two plasma plumes to connect.

**Figure 6:**
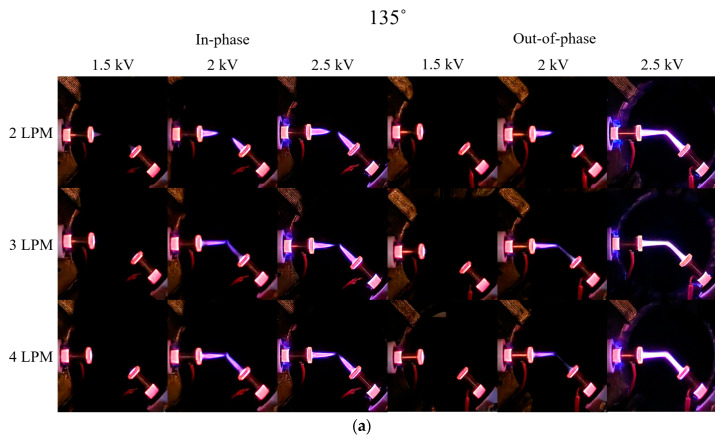

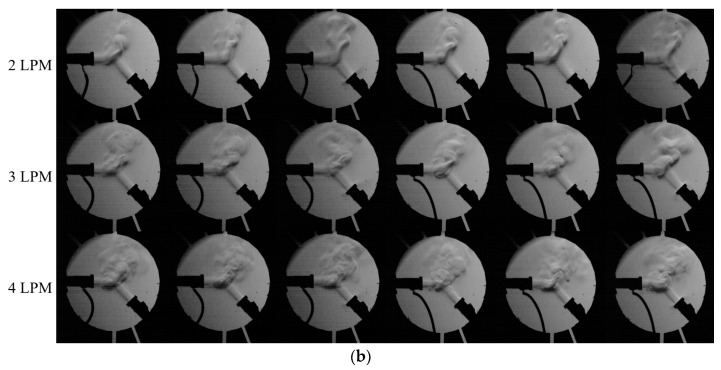
(**a**) Digital camera images and (**b**) Schlieren images for in-phase and out-of-phase configurations with two plasma jets positioned at 135°.

Figure 6 presents Schlieren and digital camera images of the in-phase and out-of-phase cases for two plasma jets positioned at a 135° angle. For the in-phase case, at 2.5 kV compared with 2 kV, the plasma plume length appeared shorter but exhibited stronger light intensity. Although an increase in voltage would typically result in a longer plasma plume, the observed decreasing length was attributed to the electrical repulsion between the plumes in the central region between the two plasma jets. Additionally, at 2 kV for the out-of-phase case, the plasma plumes at 3 and 4 LPM started to merge, unlike the 90° out-of-phase case shown in Figure 4a. When 2.5 kV was applied, the two plasma plumes merged but did not rise upward, in contrast to the behavior observed in the 90° out-of-phase case.

**Figure 7:**
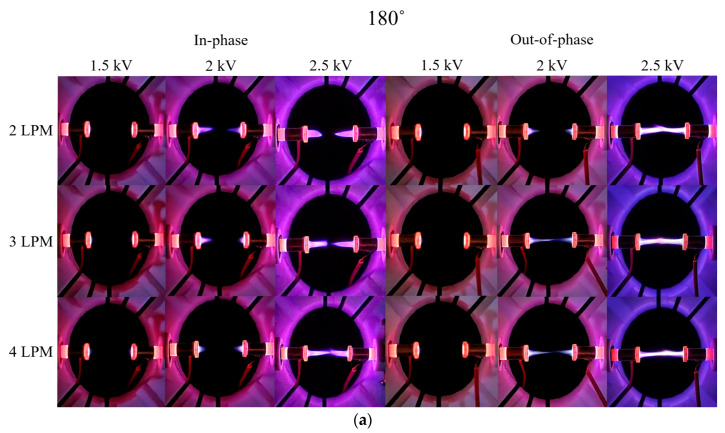

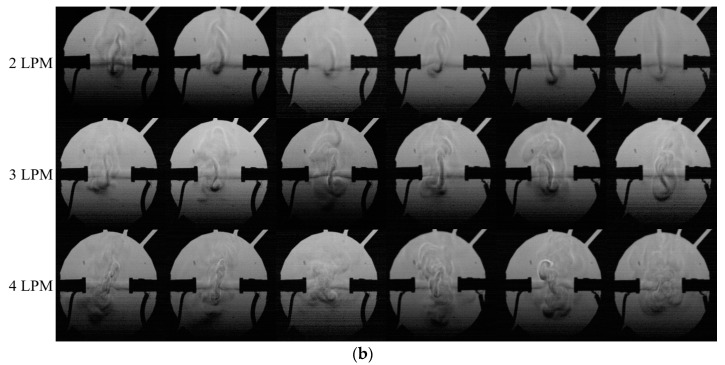
(**a**) Digital camera images and (**b**) Schlieren images for in-phase and out-of-phase configurations with two plasma jets positioned at 180°.

**Figure 8:**
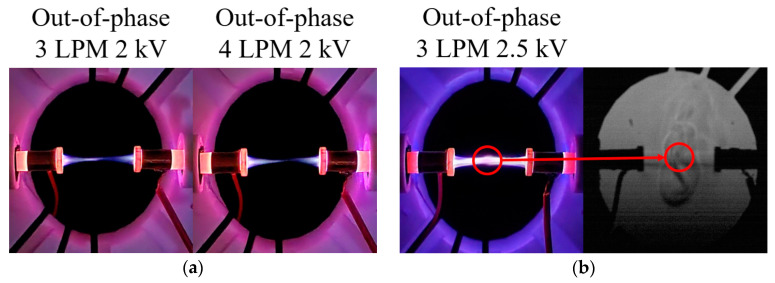
Out-of-phase case (**a**) 3, 4 LPM 2 kV digital camera images and (**b**) 3 LPM 2.5 kV digital camera image and Schlieren image positioned 180°.

Figure 7 presents digital camera and Schlieren images of the in-phase and out-of-phase cases for two plasma jets positioned at 180°. Unlike the in-phase 90° case, the plasma plume length decreases with increasing flow rate when 2 kV is applied. This phenomenon is due to the He flows from each plasma jet exerting a strong influence on the opposing jet’s flow as the flow rate increases. For the in-phase case at 4 LPM and 2.5 kV, the plasma plumes from the two jets did not merge, maintaining a very small gap, denoted as $d_{min}$. For the out-of-phase case, like the 135° out-of-phase case, weak plume merging occurred at 3 and 4 LPM when 2 kV was applied, as shown in Figure 8a. Additionally, at 2.5 kV, plume merging occurred between the two plasma jets, with a slight increase in thickness at the center. This can be observed in Figure 8b, where the Schlieren image shows the collision of flows from the two plasma jets.

Table 1 shows the applied voltage at which plasma plume merging occurred for all angles. It can be seen that the 90° case requires a relatively higher voltage to be applied for plume merging to occur compared with the 135° and 180° cases.

**Figure 9:**
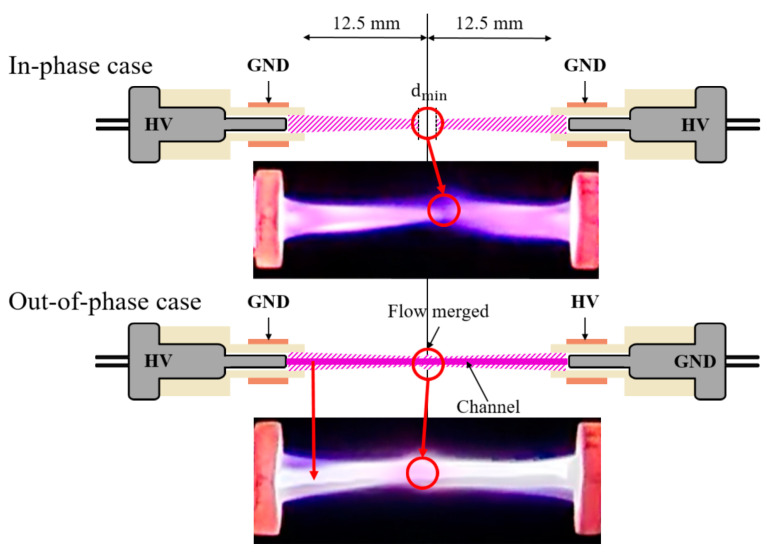
In-phase and out-of-phase case dynamics diagram.

Figure 9 illustrates the plasma plume dynamics generated by two plasma jets. For the out-of-phase case, the stainless-steel electrode of the opposing plasma jet is at ground potential relative to the high-voltage stainless steel electrode of the left plasma jet, leading to the formation of a channel between these electrodes. This channel likely facilitates the movement of seed electrons from the surrounding atmosphere, enabling collisions with He atoms and resulting in a brighter emission compared with the in-phase case. On the other hand, according to Douat et al. [30], for the in-phase case, the plasma plumes from the two jets do not merge and maintain a small gap, $d_{min}$, in a few hundred µm. In contrast, for the out-of-phase case, the plumes merge, resulting in a brighter emission. A comparison of the experimental results from Douat et al. [30] and this study confirms that two plumes do not merge for the in-phase case, and the minimum gap, $d_{min}$, is maintained. This also applies to the 90° and 135° cases.

### 3.3. Optical Emission Spectroscopy (OES)

OES measurements were taken from the left plasma jet in the 90° case due to difficulties in measuring at other angles and were normalized to the 706 nm peak value observed for the in-phase case at 2 LPM and 1.5 kV. The measurement range was 300–750 nm.

**Figure 10:**
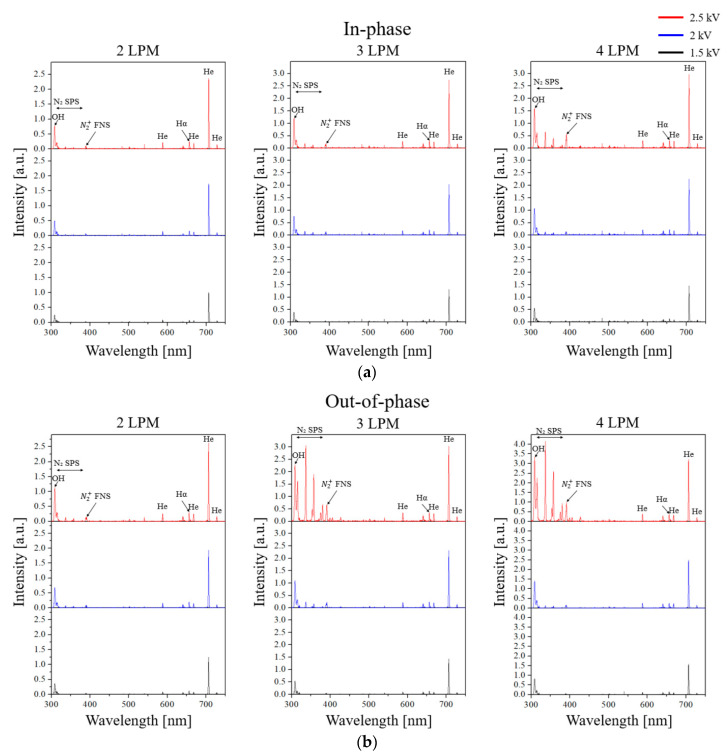
Emission spectra of (**a**) in-phase and (**b**) out-of-phase cases for the variations of the gas flow rates (2, 3, and 4 LPM) and the applied voltage (1.5, 2, and 2.5 kV).

**Figure 11:**
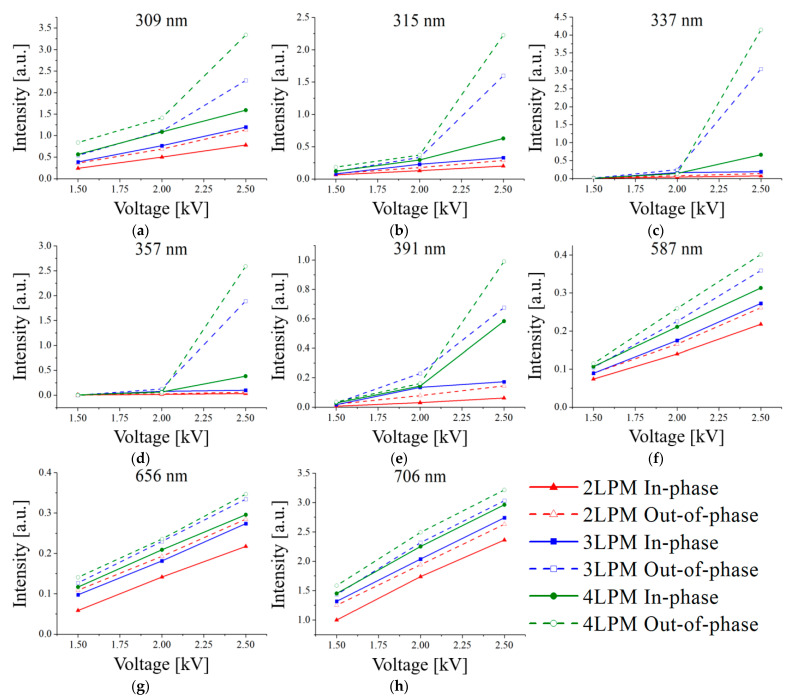
Emission intensities of (**a**) OH (309 nm), (**b**–**d**) $N_{2}$ SPS (315, 337, and 357 nm), (**e**) $N_{2}^{+}$ FNS (391 nm), and (**f**–**h**) He (587, 656, and 706 nm). Lines are for an eye-guide only.

Figure 10 presents the emission spectra as various voltage and flow rates for both in-phase and out-of-phase cases. Overall, the peak values tend to increase with rising voltage and flow rate, with the He peak at 706 nm being the most prominent. Additionally, the OES peaks of OH, the second positive system (SPS) of $N_{2}$, the first negative system (FNS) of $N_{2}^{+}$, and Hα are measured at wavelengths of 309 nm (OH), 300–400 nm ($N_{2}$ SPS, $C^{3}\Pi_{u}\to B^{3}\Pi_{g}$), 391 nm ($N_{2}^{+}$ FNS, $B^{2}\Sigma_{u}^{+}\to X^{2}\Sigma_{g}^{+}$), and 656 nm (Hα). While the emission spectra profile for the in-phase case did not show significant variation, for the out-of-phase case, the OH, $N_{2}$ SPS, and $N_{2}^{+}$ FNS peaks increase notably when 2.5 kV is applied at flow rates of 3 and 4 LPM. When 2.5 kV was applied for the out-of-phase case at 3 and 4 LPM, a channel was formed between the stainless-steel electrodes of the two plasma jets. This plume merging results in increased light intensity compared with the in-phase case. Figure 11 confirms that the light components due to the plume merging of the two plasma jets are predominantly composed of OH, $N_{2}$ SPS, $N_{2}^{+}$ FNS, and He.

Figure 11 shows the emission intensity trends for the wavelength regions corresponding to OH, $N_{2}$ SPS, $N_{2}^{+}$ FNS, and He as functions of flow rate, voltage, and phase. Generally, the emission intensity for the out-of-phase case was stronger than for the in-phase case, with intensity increasing as flow rate and voltage rose. As previously described, the 309 nm (OH) and $N_{2}$ SPS and $N_{2}^{+}$ FNS peaks within the 300–400 nm range exhibited significant changes when 2.5 kV was applied at 3 and 4 LPM for the out-of-phase case. Additionally, the emission intensities at wavelengths 587 nm (He, $3^{3}D⟶2^{3}P$), 656 nm (He, $3^{1}D⟶2^{1}P$), and 706 nm (He, $3^{3}S⟶2^{3}P$), associated with metastable He states, which showed little difference in Figure 10, increased linearly with the rise in voltage and flow rate.

**Figure 12:**
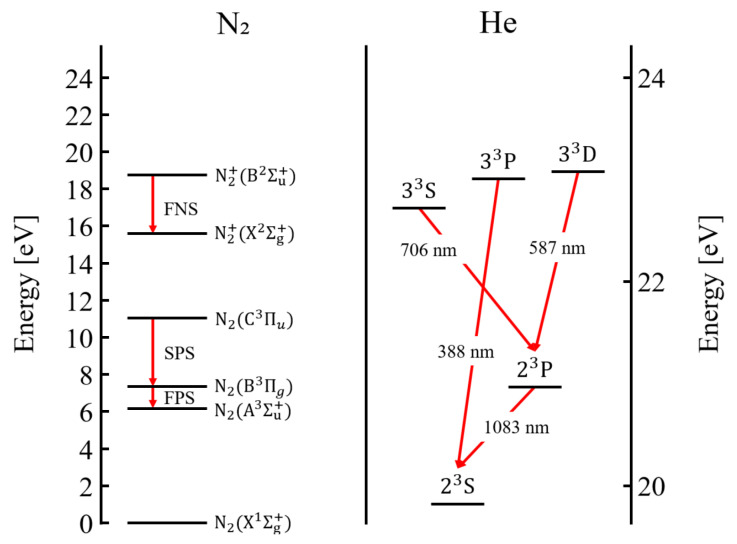
Energy level diagram for the emission spectra of $N_{2}$ and He.

Figure 12 illustrates the $N_{2}$ SPS, $N_{2}^{+}$ FNS, and He energy states [37,38,39,40,41,42]. The $N_{2}$ SPS results from electron impact excitation by Equation (1), while the FNS is caused by Penning ionization by Equation (2) and electron impact ionization by Equation (3) [43,44,45,46].

(1) $$N_{2}\left(X^{1}\Sigma_{g}^{+}\right)+e\to N_{2}\left(C^{3}\Pi_{u}\right)+e,$$

(2) $$He\left(2^{3}S\right)+N_{2}\left(X^{1}\Sigma_{g}^{+}\right)\to He+N_{2}^{+}\left(B^{2}\Sigma_{u}^{+}\right)+e,$$

(3) $$N_{2}\left(X^{1}\Sigma_{g}^{+}\right)+e\to N_{2}^{+}\left(B^{2}\Sigma_{u}^{+}\right)+2e$$

He causing Penning ionization occurs as the energy state of He transitions from $2^{3}P\to 2^{3}S$. Additionally, He in the $2^{3}P$ state is produced by He in the $3^{3}D$ (587 nm) and $3^{3}S$ (706 nm) states. Therefore, if the emission intensities at 587 nm and 706 nm are strong, it indicates a significant presence of 2S state He and the occurrence of Penning ionization. It suggests that when plume merging due to channel formation occurs, a substantial number of metastable He atoms and high-energy electrons are generated, leading to an increase in $N_{2}$ SPS and $N_{2}^{+}$ FNS reactions through collisions with nitrogen molecules in the atmosphere.

Overall, the Schlieren imaging and OES data reveal three key observations. First, for the in-phase case, plume merging does not occur and has a small gap ($d_{min}$). In contrast, plume merging is observed for the out-of-phase case. Second, when plume merging occurs, the light emission intensity increases, with a particularly significant enhancement in the emission intensities of OH, $N_{2}$ SPS, and $N_{2}^{+}$ FNS. Third, the formation of $N_{2}$ SPS and $N_{2}^{+}$ FNS is driven by high-energy electrons and metastable He states, which are accelerated through the channel. It occurs due to the metastable state He ($2^{3}S)$ generated when the seed electron is accelerated through the channel and collides with ambient He, as well as when the accelerated electron collides with atmospheric N_2_.

## 4. Discussion and Conclusions

In this study, we investigated the plasma characteristics of helium atmospheric pressure plasma jets (He APPJs) by varying the flow rate, voltage, and angle between the two APPJs. For the in-phase case, it was observed that the plasma plume length increases with increasing voltage and flow rate; however, plume merging did not occur, and a small gap $d_{min}$ was maintained. Conversely, for the out-of-phase case, plume merging was observed due to the formation of a channel. When plume merging occurred, the emission intensity increased compared with cases without merging. Additionally, optical emission spectroscopy (OES) was employed to observe the emission spectra under different voltages and flow rates in both in-phase and out-of-phase cases. In cases without plume merging, the emission spectra profiles in the 300–750 nm range were generally similar between the in-phase and out-of-phase cases. However, for the out-of-phase case where plume merging occurred, a significant increase in the peak emission intensities of OH, $N_{2}$ SPS, and $N_{2}^{+}$ FNS was observed. This phenomenon is interpreted as resulting from the interaction between metastable He, generated by accelerated seed electrons through the channel, and ambient He as well as the collision of accelerated electrons with atmospheric nitrogen. Furthermore, it is anticipated that a wider range of physical phenomena occurring in the two plasma jets can be observed by employing an ICCD camera and numerical modeling. Additionally, if a mixture gas of N_2_ or O_2_ is introduced to the two plasma jets, it is expected that the formation of reactive species could enable applications in sterilization and medical fields. Furthermore, by placing an object between the two plasma jets, it is anticipated that surface treatment rendering the material surface hydrophilic could also be achieved.

## Figures and Tables

**Table 1:** Minimum voltage for plume merging as a function of angle and flow rate.

	2 LPM	3 LPM	4 LPM
90°	2.14 kV	2.08 kV	2.04 kV
135°	2.12 kV	2 kV	2 kV
180°	2.12 kV	2 kV	2 kV

## Data Availability

The data sets generated during and/or analyzed during the current study are available from the corresponding author upon reasonable request. The data set is stored on the local Pusan National University server.
